# An episode-based cost analysis of virtual-first versus in-person-first care to treat common acute conditions among members of a large national payor

**DOI:** 10.1186/s12913-025-13154-1

**Published:** 2025-07-29

**Authors:** Amanda L. Zaleski, Xinbei Guan, Kelly J. Thomas Craig, Christopher Junk, Arthur T. McGill, Henry Gordon, Dorothea J. Verbrugge, Kristofer Caya

**Affiliations:** 1 Clinical Evidence Development, Aetna® Medical Affairs, CVS Health®, Wellesley, MA USA; 2 Analytics & Behavior Change, CVS Health, Wellesley, MA USA; 3 Aetna Network Virtual Care, CVS Health, Wellesley, MA USA

**Keywords:** Primary care, Telemedicine, Telehealth, Care delivery, virtual care

## Abstract

**Background:**

The potential of virtual care as an alternative to in-person visits is promising, yet its economic impact is insufficiently understood. This evaluation represents an episode-based, cost impact analysis of virtual-first (versus in-person first) care to treat the most prevalent primary care acute conditions among Medicare Advantage (MA) and commercial fully-insured (C-FI) members of a large national health plan in the United States.

**Methods:**

Retrospective episodes-of-care and medical claims analyses identified members (*N* = 366,195; MA: 126,363, C-FI: 239,832) with resolved, pre-specified, acute primary care episodes (*N* = 455,231; MA: 141,034, C-FI: 314,197) between 1/1/2022–6/30/2022. Propensity score weighting estimated % difference in healthcare expenditures between virtual-first episodes and an adjusted cohort of in-person-first episodes.

**Results:**

Within the MA cohort, 7.6% (range: 0.7–24.8%) of episodes utilized virtual-first care with observed cost-of-episode 10–24% lower than in-person-first care for 6 of 11 included conditions (all *P* < 0.05), including: otolaryngology disease (-24 ± 2%), rhinitis (-20 ± 4%), gastroenterology disease (-20 ± 7%), minor bacterial skin infections (-17 ± 7%), sinusitis (-14 ± 4%), and bronchitis (-11 ± 4%). Within the C-FI cohort, 12.6% (range: 2.8–40.4%) of episodes utilized virtual-first care with observed cost-of-episode 9–33% lower than in-person-first care (all *P* < 0.001) for 12 of 16 included conditions (all *P* < 0.001), including: urinary tract infection (-33 ± 5%), viral skin infection (-29 ± 6%), gastroenterology disease (-27 ± 5%), rhinitis (-28 ± 5%), otolaryngology disease (-25 ± 2%), sinusitis (-25 ± 2%), urological disease (-23 ± 9%), contact dermatitis (-19 ± 5%), viral pneumonia (-17 ± 12%), bronchitis (-15 ± 4%), fungal skin infection (-11 ± 6%), and minor bacterial skin infection (-9 ± 7%), and 4 ± 2% higher to treat exposure to infectious disease (*P* = 0.001). There were no between-group differences in cost-of-episode to treat: skin inflammation (MA & C-FI), urinary tract infection (MA), exposure to infectious disease (MA), fungal skin infection (MA), low back pain (C-FI), or migraine headache (C-FI) (all *P* > 0.081).

**Conclusion:**

This real-world study of a large national sample of geographically diverse members demonstrates the potential of virtual-first care to resolve acute conditions at lower cost compared to in-person-first care. The use of episode-based analytical tools enhances the significance of these findings by enabling a proxy for clinical outcomes.

## Introduction

In the wake of the global COVID-19 pandemic, the healthcare landscape underwent rapid and transformative changes to ensure the provision of essential medical services while mitigating the risks associated with in-person interactions [1]. Central to this transformation was the widespread adoption of virtual care technologies; largely driven by necessity and regulatory changes that expanded use of virtual care [1, 2]. These technological and regulatory advancements not only addressed the immediate challenges posed by the pandemic but also presented an opportunity to revolutionize the future of healthcare delivery. While existing literature underscores the potential benefits of virtual-first care in enhancing timely access to care, a comprehensive and multi-dimensional evaluation of its impact on clinical and economic outcomes remains a critical research gap [3, 4].

Payors are a critical stakeholder in defining reimbursement patterns, coverage policies, and cost-sharing arrangements for virtual care [2]. The interplay between virtual care, payors, and the broader healthcare ecosystem represents a key opportunity for future incentivization and sustainability of virtual care adoption [5]. Moreover, access to real-world data sources, such as claims-based data, offers a unique vantage point that captures the complexities and subtleties of actual patient experiences and healthcare practices. Leveraging real-world data, the application of advanced analytical capabilities contributes to a more holistic and ecologically valid understanding of how virtual care strategies impact clinical outcomes, healthcare utilization, and cost dynamics within the evolving landscape of the COVID-19 pandemic and beyond.

However, to fully understand the economic impact of virtual-first care, it is important to move beyond the limitations of traditional encounter-based cost analyses, which often overlook the holistic trajectory of a disease episode and can be diluted by high treatment costs and duration of chronic conditions. Unlike traditional approaches, episode-based analytical methodology captures the full spectrum of healthcare utilization from diagnosis to recovery for one “episode” of an acute illness; serving as a valuable surrogate for clinical quality for illnesses that resolve within a defined timeframe (i.e., acute conditions) [6].

Thus, the primary goal of this evaluation was to characterize the real-world cost impact of virtual care as the initial modality of care (i.e., virtual-first care) for treating top prevalent, resolved acute primary care conditions (compared to in-person-first care) among members of a large United States (US)-based national health plan.

## Methods

### Study design

This study deployed a propensity-weighted, two-group, observational cohort design. Briefly, retrospective claims analyses of commercial fully-insured (C-FI) and Medicare Advantage (MA) members enrolled for ≥ 6 months prior to the episode start date identified resolved acute primary care episodes starting between January 1, 2022, to June 30, 2022.

Administrative medical claims data included diagnosis codes, procedures, laboratory encounters, sites of care, provider information, and service costs. Claims data also included aggregations of the above information in the forms of medical cases, episode treatment groups, chronic condition flags, and predictive risk scores. Demographic information collected during health insurance enrollment included self-reported sex, age, race, ethnicity, location, plan benefit details, and census tract statistics. All demographic and administrative medical claims data were deidentified, aggregated, and analyzed.

Propensity score weighting was used to estimate the percent difference in healthcare expenditures between virtual-first episodes and a cohort of in-person-first episodes adjusted for member characteristics and healthcare utilization.

### Study population

All participants were MA and C-FI enrollees of a large national health plan. Inclusion criteria included (1) continuous health plan eligibility throughout the study evaluation period, (2) ≥ 1 resolved pre-specified, acute primary care episode within the study evaluation period (i.e., January 1, 2022 to June 30, 2022), and (3) administrative claims data for ≥ 6 months preceding the episode start date. Members with episodes involving inpatient services and/or emergency department as initial encounters were excluded.

The virtual-first care group included members who received virtual-first care for an acute treatment episode. The comparison group (i.e., in-person-first group) included health plan members who were active enrollees during the same study evaluation time period but received traditional, in-person-first care. Note that members with more than one treatment episode were coded as unique members (i.e., not double counted).

### Ethics approval

This study was reviewed and determined to be exempt by the Sterling Institutional Review Board (#11260) under 45 CFR 46.104(d)(4). The research was conducted in accordance with the ethical principles outlined in the Declaration of Helsinki. A waiver of Health Insurance Portability and Accountability Act authorization was obtained for the use and disclosure of aggregated, deidentified member data. No compensation was provided.

### Selection of included acute primary care episodes

Application of a single, licensed, proprietary episode grouper algorithm (Optum^®^ Symmetry^®^ Episode Treatment Groups [ETG^®^]) classified clinically relevant and condition-specific episodes-of-care. Retrospective analysis of claims data identified most prevalent acute episodes treated in a primary care setting from the preceding year (i.e., January 1, 2021, to December 31, 2021) for both payor cohorts. Most prevalent episodes were appraised by an internal team of subject matter experts and medical directors and refined based on clinical gestalt, resulting in a final list of acute primary care episodes of interest for the MA and C-FI cohort, each. The final list of pre-specified episodes of interest for the MA cohort (*n* = 12), included: (1) acute bronchitis; (2) acute sinusitis; (3) skin inflammation; (4) urinary tract infection; (5) otolaryngology disease; (6) rhinitis; (7) viral pneumonia; (8) fungal skin infection; (9) minor bacterial skin infection; (10) exposure to infectious disease; 11) gastroenterology disease, and 12) bacterial lung infection. The final list of episodes of interest for the C-FI cohort (*n* = 17) included all episodes previously listed (i.e., MA cohort), in addition to five (5) additional episodes, including: low back pain; migraine headache; contact dermatitis; viral skin infection; and urological disease. Of note, bacterial lung infection was not included in the final cost impact analysis due to low volume of episodes observed in the evaluation period, resulting in a total of 11 and 16 episodes evaluated for the MA and C-FI cohorts, respectively.

### Statistical analysis

Retrospective analysis of claims data was used to identify members with resolved pre-specified, acute primary care episodes for the study evaluation period (i.e., January 1, 2022, to June 30, 2022). In the absence of randomized exposure to treatment, propensity score weighting was deployed to balance potential confounders that may obfuscate the impact of virtual care on the cost of acute care. More specifically, a logistic regression selection model was estimated to generate propensity scores for the start of virtual-first episodes. Overlap weighting was used to adjust the weight of each observation in measuring the impact of virtual care. Overlap weighting assigns a weight to each observation that is equal to the probability of that observation belonging to the opposite class that was observed [7]. For example, if P is equal to the propensity for a member to have a virtual-first visit then members with in-person first episodes will be weighted with P and members with virtual-first episodes will be weighted with (1-P). This methodology simulates a randomized clinical trial by balancing confounders post-weighting and separately reduces the impact of outlier observations [7]. More than 100 member features, including, but not limited to sociodemographic factors, healthcare utilization patterns, proprietary risk scores, chronic condition diagnoses, and social determinants of health were used in the selection and outcome models to obtain doubly robust estimates.

Claims data were used to compute average medical spend for resolved episodes-of-care across each subcategory (i.e., primary care acute condition) for virtual-first and in-person-first care groups in US dollars (proprietary; data not shown). Difference in healthcare spend (i.e., cost-of-episode) was calculated as virtual-first spend minus in-person-first spend, adjusting for confounders listed above, and presented as a % difference. Negative values indicate lower spend for virtual-first care compared to in-person-first care.

A planned sensitivity analysis was conducted using various causal inference methods and outcome feature selection models established a priori, including targeted maximum likelihood estimation, augmented inverse probability weighting, and propensity score weighting with overlap weights. Robustness checks were conducted to check the sensitivity of results to model specifications. Robustness checks include alternative specifications of the selection model, application of doubly robust modeling techniques by also specifying confounders in the outcome model and testing different weighting approaches such as inverse propensity of treatment weighting. Of note, the preliminary results remained robust to various statistical models (data available upon request).

Two-tailed *T*-tests were used to test for potential differences in member characteristics (i.e., age, sex, geography, condition prevalence, and healthcare utilization) between virtual-first and in-person-first groups for both payor cohorts (i.e., MA and C-FI). Statistical significance was defined as *P* < 0.05.

## Results

Table 1 details sociodemographic and clinical characteristics segmented by payor cohort (i.e., MA and C-FI) and care delivery group (i.e., virtual-first care vs. in-person-first care). Among the MA cohort (*N* = 126,363) there were 141,034 resolved acute primary care episodes of which 10,820 were virtual-first episodes and compared to a cohort of in-person-first episodes (*N* = 130,214). Among the C-FI cohort (*N* = 239,832) there were 314,197 resolved acute primary care episodes of which 39,453 were virtual-first episodes and compared to a cohort of 274,744 in-person-first episodes.

**Table 1:** Sociodemographic and clinical characteristics segmented by payor cohort and care delivery group

Characteristic	Medicare Advantage (*N* = 126,363)		Commercial FI (*N* = 239,832)	
	Virtual-First	In-Person-First	Virtual-First	In-Person-First
Episodes (*n*)	10,820	130,214	39,453	274,744
Mean age ± SD (years)	70.88 ± 9.56***	72.91 ± 9.79	36.00 ± 7.27***	32.94 ± 19.39
Sex (%)				
Male	34.24***	39.93 ± 48.97	39.14***	44.15
Female	65.76***	60.07	60.85***	55.83
Unknown/Missing	0.0 ± 0.0	0.0 ± 0.0	0.0085*	0.02
Race (%)				
White	77.07***	81.60	46.97***	43.13
Black	12.54***	10.86	6.61	6.83
Asian	2.35**	1.82	5.61	5.53
American Indian/Alaskan Native	0.20	0.21	0.14	0.17
Native Hawaiian/Pacific Islander	0.00	0.00	0.1	0.08
Hispanic/Latino	3.59	2.03	7.84***	6.71
Other	1.92	1.40	4.57**	4.19
Unknown	2.31	2.09	28.17***	33.36
Location (%)				
Urban	31.12***	24.12	47.41	45.06
Suburban	25.12	24.92	23.54	23.80
Rural	43.76***	50.95	29.05***	31.14
Chronic conditions (%)				
1	5.06	5.24	22.09	21.67
≥ 2	91.92*	91.25	36.08***	31.87

*Abbreviations*: *FI* fully-insured, *SD* standard deviation Between-group difference for virtual-first versus in-person first

*P < 0.05; **P < 0.01; ***P < 0.001

Sum total, the study population (*N* = 366,195 members; *N* = 455,231 episodes) was comprised of female (57.9%) and male (42.1%) adults across the lifespan (mean age: 46.8 ± 24.9 years; range: 0 to ≥ 90 years [suppressed for Safe Harbor de-identification]) residing across urban (38.3%), rural (37.5%), and suburban (24.2%) areas across the US.

### MA cohort characteristics

On average, the virtual-first care group was comprised of mostly White (77.1%), female (65.8%), older adults (mean age: 70.9 ± 9.6 years), of which a majority (91.9%) had ≥ 2 chronic conditions. The virtual-first care group was less likely to reside in rural locations and more likely to reside in urban locations (*P* < 0.001). In addition, there were marginal differences in demographic characteristics such that the virtual-first care group were slightly younger; more likely to be female; and with higher proportion of non-White populations (all *P* < 0.001).

### C-FI cohort characteristics

On average, the virtual-first care group was comprised of mostly White (47.0%), female (60.9%), young to middle-aged adults (mean age: 36.0 ± 7.3 years), of which 31.9% had ≥ 2 chronic conditions. The virtual-first-care group was less likely to reside in rural locations and more likely to reside in urban locations (*P* < 0.001). In addition, there were marginal differences in demographic and clinical characteristics such that the virtual-first care group was slightly older; more likely to be female or unknown gender, White, Hispanic, or other races; and more likely to have ≥ 2 chronic conditions (all *P* < 0.01).

### MA cohort outcomes

**Fig. 1:**
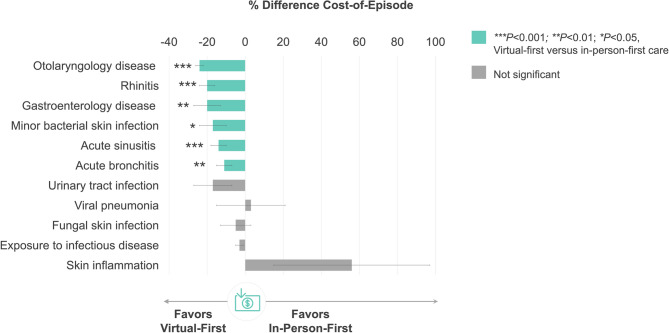
Between-Group Difference in Healthcare Expenditures by Acute Condition in the Medicare Advantage Cohort. Figure legend: % difference calculated as virtual-first care minus in-person-first care such that negative values indicate lower spend for virtual-first care compared to in-person-first care

Among the most commonly treated acute episodes, 7.6% (range: 0.7–24.8%) utilized virtual-first care (Table 2). Figure 1 displays % difference in healthcare expenditures for evaluated acute conditions. When treated through virtual-first care, cost-of-episode was 10–24% lower than in-person-first care (all *P* < 0.05) for 6 of 11 included conditions (all *P* < 0.05), including: otolaryngology disease (−24 ± 2%, *P* = 0.000), rhinitis (−20 ± 4%, *P* = 0.000), gastroenterology disease (−20 ± 7%, *P* = 0.003), minor bacterial skin infection (−17 ± 7%, *P* = 0.017), sinusitis (−14 ± 4%, *P* = 0.000), and bronchitis (−11 ± 4%, *P* = 0.003).

**Table 2:** Top acute conditions for virtual-first care among Medicare Advantage members

Episode Diagnosis^a^	Members (*N*)	Episodes (*N*)	Virtual-First Care Visit (%)
Exposure to infectious disease	32,070	37,600	8.8
Otolaryngology disease	9,524	9,583	24.8
Acute sinusitis	6,067	6,149	18.8
Acute bronchitis	5,644	5,730	19.2
Rhinitis	13,504	13,591	7.9
Gastroenterology disease	8,775	8,852	6.7
Urinary tract infection	6,041	6,213	5.7
Minor bacterial skin infection	10,416	10,629	2.5
Fungal skin infection	30,155	32,012	0.7
Viral pneumonia	1,586	1,590	13.3
Skin Inflammation	8,712	8,842	1.6
Bacterial lung infection	242	243	6.2

^a^Ranked by volume (greatest to least) of virtual-first care episodes during the evaluation timeframe

There were no between-group differences in cost-of-episode for urinary tract infection (*P* = 0.081), exposure to infectious disease (*P* = 0.106), skin inflammation (*P* = 0.172), or fungal skin infection (*P* = 0.518).

### C-FI cohort outcomes

**Fig. 2:**
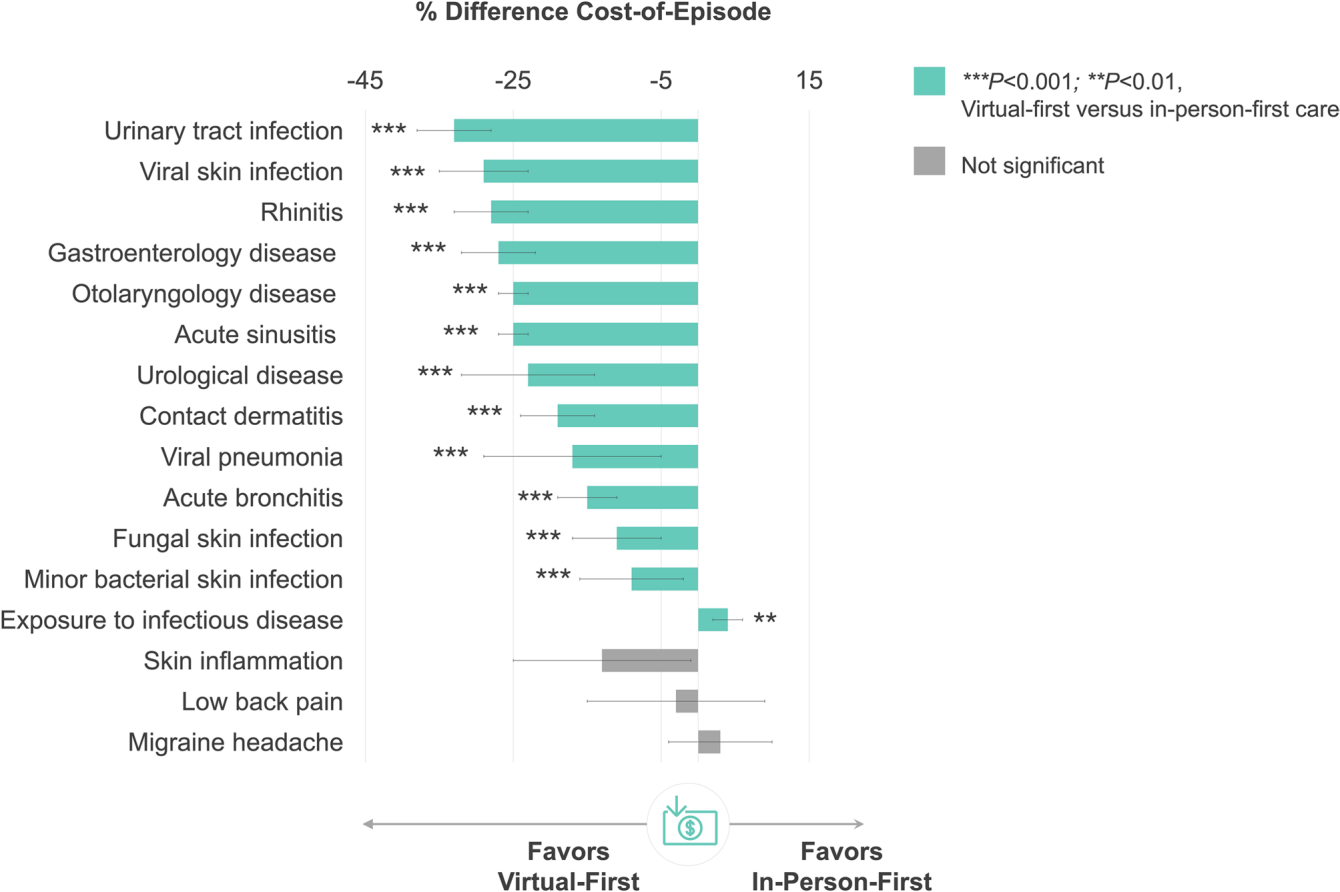
Between-Group Difference in Healthcare Expenditures by Acute Condition in the Commercial Fully-Insured Cohort. Figure legend: % difference calculated as virtual-first care minus in-person-first care such that negative values indicate lower spend for virtual-first care compared to in-person-first care

Among the most commonly treated acute episodes, 12.6% (range: 2.8–40.4%) utilized virtual-first care (Table 3). Figure 2 displays % difference in healthcare expenditures for evaluated acute conditions. When treated through virtual-first care, cost-of-episode was 9–33% lower than in-person-first care (all *P* < 0.001) for 12 of 16 included conditions (all *P* < 0.001), including: urinary tract infection (−33 ± 5%, *P* = 0.000), viral skin infection (−29 ± 6%, *P* = 0.000), gastroenterology disease (−27 ± 5%, *P* = 0.000), rhinitis (−28 ± 5%, *P* = 0.000), otolaryngology disease (−25 ± 2%, *P* = 0.000), sinusitis (−25 ± 2%, *P* = 0.000), urological disease (−23 ± 9%, *P* = 0.000), contact dermatitis (−19 ± 5%, *P* = 0.000), viral pneumonia (−17 ± 12%, *P* = 0.000), bronchitis (−15 ± 4%, *P* = 0.000), fungal skin infection (−11 ± 6%, *P* = 0.000), and minor bacterial skin infection (−9 ± 7%, *P* = 0.000).

**Table 3:** Top acute conditions for virtual-first care among commercial fully-insured members

Episode Diagnosis^a^	Members (*N*)	Episodes (*N*)	Virtual First Care Visit (%)
Exposure to infectious disease	106,056	136,881	9.6
Otolaryngology disease	19,824	20,064	28.1
Acute sinusitis	9,530	9,640	40.4
Gastroenterology disease	14,324	14,493	16.6
Rhinitis	21,009	21,156	9.6
Migraine headache	9,806	10,400	19.4
Acute bronchitis	5,964	6,050	31.7
Urinary tract infection	4,914	5,054	30.9
Skin inflammation	16,750	17,241	8.4
Minor bacterial skin infection	10,247	10,524	10.4
Contact dermatitis	6,134	6,192	14.0
Viral pneumonia	6,167	6,173	12.1
Urological disease	7,201	7,296	10.2
Fungal skin infection	10,546	11,119	6.2
Low back pain	10,778	23,250	2.8
Viral skin infection	8,263	8,462	7.4
Bacterial lung infection	200	202	15.3

^a^Ranked by volume (greatest to least) of virtual-first care episodes during the evaluation timeframe

Cost-of-episode was 4 ± 2% higher for virtual-first care to treat exposure to infectious disease (*P* = 0.001).

There were no between-group differences in cost-of-episode for skin inflammation (*P* = 0.296), low back pain (*P* = 0.823), or migraine headache (*P* = 0.334).

## Discussion

### Principal findings

This study sought to conduct a retrospective, episode-based cost analysis of virtual-first versus in-person-first care to treat the most prevalent acute conditions among 366,195 MA and C-FI members of a large national payor. By leveraging claims-based data within the episode grouper framework to assess the impact of virtual-first care encounters, this research aimed to contribute meaningful insights to inform strategic decision-making, improve the patient and provider experience, and foster equitable access to effective and affordable health and healthcare. Major findings were that cost-of-episode for treatment with virtual-first care was markedly lower than in-person-first care for a substantial proportion of evaluated conditions. These trends were consistent across both payor cohorts with virtual-first care expenditures 10 to 24% and 9 to 33% lower for a majority of conditions commonly treated in the MA and C-FI population, respectively; highlighting the cost-saving potential across diverse payor populations. 

### Interpretation of principal findings

These results underscore the viability of virtual care as a lower-cost alternative to traditional in-person visits; particularly for the initial care delivery modality for certain acute conditions whereby virtual-first care consistently demonstrated lower cost-of-episode to treat to resolution. Specifically, cost-of-episode for virtual-first care was consistently more favorable than in-person-first care for the treatment of otolaryngology disease, bronchitis, sinusitis, rhinitis, minor bacterial skin infection, and gastroenterology disease in both MA and C-FI cohorts.

Notably, cost-of-episode was 4% higher for virtual-first care to treat exposure to infectious disease (*P* < 0.001) in the C-FI cohort and tended to be -3% lower in the MA cohort, though this did not achieve statistical significance (*P* = 0.106). This finding corroborates with a peak in COVID-19 cases during the evaluation timeframe (January 1, 2022 to June 30, 2022) [8]. This period likely influenced healthcare utilization and reimbursement patterns as virtual care was widely used for triage and ongoing symptom monitoring. It is possible that virtual-first care in this context led to increased downstream services, including confirmatory testing, additional visits, and return-to-work clearance; particularly in commercial populations where many employers required documentation, however, this is purely speculative. Despite observed variations in cost-of-episode, the overall benefits of virtual-first care in the context of a public health pandemic should be carefully weighed against these marginal differences.

It is interesting to note that, with the exception of exposure to infectious disease, there were no other scenarios where virtual-first care was more costly than in-person-first care. Although not designed as an inferiority study, these findings are promising and suggest virtual-first care to be a lower-cost modality to effectively treat a diverse set of the most commonly experienced acute primary care conditions. Nevertheless, the lack of between-group difference in cost-of-episode for other select conditions could signal how virtual-first care might be selectively deployed to optimize value-based care for certain populations, conditions, or healthcare settings.

For example, there were no between-group differences in healthcare expenditures for the treatment of skin inflammation across both cohorts (i.e., MA and C-FI). Within the MA cohort only, there were no observed between-group differences for the treatment of viral pneumonia and fungal skin infection (compared to -17% and -11% lower spend in the C-FI cohort, respectively). Additionally, virtual-first care to treat urinary tract infection tended to be -17% lower (*P*= 0.081) than in-person-first care in the MA cohort (compared to -33% lower in the C-FI cohort), though this did not achieve statistical significance. Within the C-FI cohort only, there were no observed between-group differences in cost-of-episode for the treatment of low back pain or migraine headache (both *P *> 0.334); both conditions in which treatment outcomes can be subject to heterogeneity [9, 10]. 

### Limitations

In addition, this study adds to the body of literature with its evaluation of real-world data of 455,231 acute primary care episodes derived from numerous platforms, providers, and patients. Access to member claims data enables quantification of real-world cost differences without relying on simulated models or estimates. Finally, the study cohort is a representative population for whom this care delivery modality would be used by (i.e., healthcare consumers belonging to a large, national health plan). As the use of virtual care platforms and their functionality continue to evolve, this study provides a benchmark for expected differences in cost-of-episode for virtual-first care delivery. There are few noteworthy limitations to this study. This study infers cost differences to be driven by virtual-first care. Although these findings were statistically significant, this study was not designed to explore a definitive causal relationship. Randomized-controlled trials are the gold-standard approach to measure the causal effects of an intervention but are not feasible to conduct for health plan–sponsored benefits. Retrospective observational studies are a statistically valid approach to test the real-world effectiveness of low-risk interventions in large, heterogenous populations [11]. Although subject to selection bias and confounding variables, propensity score weighting methods allows for the comparability of pre-intervention covariates and control for any potential confounding bias in reported outcomes [7, 12]. In addition, this study was not designed to conduct a cost category analysis to characterize between-group differences in healthcare expenditures. However, preliminary post hoc analysis revealed consistent trends across payor cohorts. Overall decreased expenditures observed in the virtual-first care delivery group appeared to be primarily attributed to decreased spend for specific sub-categories of medical costs, including: primary care, specialist care, and use of ancillary services such as imaging and laboratory encounters. Although interesting, we caution that these findings are preliminary and are a research priority for future evaluations. Lastly, administrative claims data are subject to limitations, including potential coding inaccuracies, missing data, and lack of clinical granularity. However, the use of episode-based grouping mitigates some of these concerns by anchoring analyses to standardized condition definitions and care trajectories, reducing dependence on single-point or potentially miscoded diagnostic codes.

Despite the noted limitations, this study possesses several strengths. This evaluation represents a rigorously designed retrospective, propensity score weighted cohort study to explore healthcare spending in a large, geographically diverse sample size of 366,195 individuals. To the best of our knowledge, this study is among the first to examine the cost impact of virtual-first versus in-person-first care for the treatment of commonly experienced acute conditions among MA and commercial fully-insured members. A majority of evidence to date has focused on chronic disease management [13, 14] or condition-specific digital health solutions [15], often without direct comparisons to in-person-first care. In addition, many studies lack clear definitions of “virtual-first” versus general telehealth use and frequently focus on care sequence rather than clinical or economic outcomes [16, 17]. A major strength of this study is the use of a single, episode grouper algorithm. This method enables a more accurate and holistic comparison of both clinical outcomes and cost between different care delivery modalities. 

### Implications for practice 

Virtual-first care holds great promise to address the quintuple aim of healthcare to: a) improve population health, b) enhance the care experience, c) enhance care team well-being, d) reduce costs or prevent loss of revenue; and e) advance health equity [18]. These data directly demonstrate the ability of virtual-first care to deliver scaled access to care for the treatment of common acute conditions with equitable clinical outcomes and for lower the cost. In addition, virtual-first care has strong potential to enhance provider experience by reducing the burden of in-person visits, potential increasing overall provider capacity. In the wake of the COVID-19 pandemic, virtual care is often promoted as a safer alternative to in-person care by minimizing the risk of infection transmission for patients and providers. Taken altogether, when appropriate, the shift from in-person-first to virtual-first care can enable healthcare professionals to manage a higher volume of patients safely and efficiently, translating to improved access to low cost, high value care for their patients. Lastly, these real-world data reflect actual member and patient experiences and preferences for care delivery modality, suggesting that virtual-first care may favorably augment the patient experience by offering more convenient and accessible care options in their preferred setting. 

## Conclusions

This real-world study demonstrates the potential of virtual-first care as a lower-cost modality for the treatment of common acute conditions in a large sample of geographically diverse MA and C-FI members across the nation. The use of episode-based analytical tools enhances the significance of these findings, as this methodology provides a proxy for clinical outcomes, which offers a more comprehensive understanding of the implications and benefits associated with virtual-first care for the treatment of common acute conditions.

## Data Availability

The data sets generated during and/or analyzed during the current study are not publicly available due to commercial data use agreements.

## Abbreviations

C-FI: commercial fully-insured
ETG®: Optum® Symmetry® Episode Treatment Groups
FI: fully-insured
MA: Medicare Advantage
SD: standard deviation
